# The Potential Effects of a Biofeedback Writing Exercise on Radial Artery Blood Flow and Neck Mobility

**Published:** 2009-06

**Authors:** Rob L. Krullaards, Johan J. M. Pel, Chris J. Snijders, Gert-Jan Kleinrensink

**Affiliations:** *Department of Neuroscience, Erasmus MC, Rotterdam, the Netherlands*

**Keywords:** radial artery, blood flow, cervical range of motion, neck trauma, biofeedback

## Abstract

**Background:**

It has been suggested that sustained contraction of the deep neck muscles may reduce axial cervical range of motion (CROM) and radial artery blood flow velocity (v_rad.art.mean_). No studies have reported both phenomena in relation to acute hand, shoulder or neck trauma.

**Procedures:**

The CROM and v_rad.art.mean_ were measured in 20 police officers prior to and immediately after a 2-hours drive on a motorcycle and immediately after a 1-minute writing exercise using biofeedback. The CROM was measured using separate inclinometers and the v_rad.art.mean_ was measured in both arms just proximal to the wrist using echo-Doppler.

**Findings:**

During the study, one officer had a motorcycle accident resulting in acute symptoms of neck trauma. His v_rad.art.mean_ was acutely reduced by 73% (right arm) and 45% (left arm). Writing with biofeedback increased his v_rad.art.mean_ by 150% (right arm) and 80% (left arm). In the remaining 19 officers, the CROM to the right was significantly increased after the 2-hours driving task (p<0.05; paired subject t-test). Writing with biofeedback increased their CROM in both directions and v_rad.art.mean_ in both arms (p<001).

**Conclusions:**

A 2-hours drive showed modest physical changes in the upper extremities. Biofeedback in writing tasks might relate to the influence of relaxation and diverting attention for neck mobility and arterial blood flow improvement.

## INTRODUCTION

Thoracic outlet syndrome is described as a group of distinct disorders resulting from compression of neural and/or vascular structures of the upper extremity at the thoracic outlet (1). It affects the brachial plexus (nerves that pass into the arms from the neck), and/or the subclavian artery and vein (blood vessels that pass between the chest and upper extremity). The brachial plexus and subclavian artery pass between the anterior and middle scalene muscles and the subclavian vein passes anteriorly to the anterior scalene as it crosses over the first rib. This region is also known as the interscalene triangle. A set of symptoms including pain in the upper extremities, unilateral sensory disturbance of the upper limb, numbness and tingling along the ulnar border of the forearm may exist due to increased compression on the brachial plexus and on subclavian vessels passing the interscalene triangle (2, 3, 4). It was suggested that contraction of the deep neck muscles like the scalene muscles and/or the longus colli muscle could cause narrowing of the triangle and hence decrease in subclavian artery and vein blood flow and decrease in neck mobility (5). Measurement of these phenomena in relation to acute hand, shoulder or neck trauma has not been reported up to date.

It was previously shown that driving a surveillance vehicle or motorcycle causes shoulder, arm and neck complaints as a result of total body vibrations (6, 7, 8). They are exposed to cold weather and may experience increased tension in hands and arms or physical stress due to awkward positioning of the upper body. The driving time and the occupational stress also correlated with the severity of the complaints. In addition, pain, numbness and stiffness in upper and lower extremities were reported (9). An adjustable steering wheel, automatic gear or cruise control improved body posture and resulted in a decrease of sick leave caused by shoulder and neck complaints (10). However, it was shown that diagnosis in the neck and shoulder region is often restricted due to a normal defensive reaction of muscle tension (11, 12, 13). This especially regards cases in which imaging techniques did not show lesions (14, 15).

In the present study, we aimed at assessment of acute increased upper extremity tension in 20 police motorcycle officers after a 2-hours drive. We focussed on the cervical range of motion and the blood flow in both arms as a measure for the build up neck and shoulder muscle tension. We hypothesised that values of both parameters decrease after a 2-hours drive on a motorcycle. To initiate an acute decrease in neck, shoulder and arm tension, we asked all police officers to do a 1-minute writing exercise using a biofeedback pen. Thus, each officer was instructed to keep a loose grip to ensure that muscle tension in hand and forearm was kept at a low level. We hypothesised that biofeedback may improve scalene muscle relaxation, leading to increase of neck mobility and blood flow. We measured the cervical range of motion and the radial artery blood flow velocity prior to and immediately after driving the motorcycle and immediately after the 1-minute writing exercise in all 20 subjects.

## METHODS

### Selection and description of participants

The subjects were 20 employees, age 40 (8) years; mean (Standard Deviation), of the Police Force Haaglanden, the Netherlands. The officers had an average body mass index of 25 (3) and worked 34.7 (7.0) hours a week. Included were healthy police motorcycle officers who met the inclusion criteria and all gave their informed consent. The study adhered to the Declaration of Helsinki for research involving human subjects.

### Technical information

**Figure 1:**
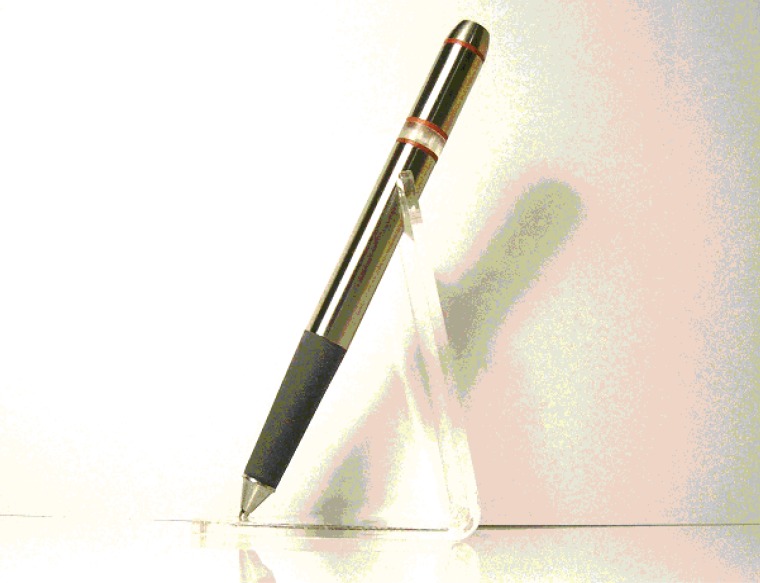
The biofeedback pen, consisting of a pressure sensor with a red light at the end of the shaft. When too much hand force is applied during writing, the red light signals the subject to loosen the grip.

Muscular resistance and joint resistance limit the rotation of the head. In the present study, we assessed the maximum head rotation until the muscular restriction was reached. To distinguish between muscular and joint resistance, all measurements were done by the same experienced physiotherapist. The head rotation was assessed with a CROM (Cervical Range of Motion) device (Performance Attainment Systems of Roseville, Minnesota). This device measured the CROM for flexion, extension, lateral flexion and left-right axial rotation using separate inclinometers (16). In this study, we only assessed bilateral axial rotation of the neck. The mean blood flow velocity of the radial artery, v_rad.art.mean_, was measured in both arms just proximal to the wrist. Measurements were performed using a Versalab (Nicolet Vascular Versalab, serial number: SEC0106); a small echo-Doppler device that detects velocity profiles in arties and veins. The 8 MHz probe was held at an angle of 45 degrees to the skin surface. This position was secured using a simple probe holder. The 1-minute writing exercise was done using a specially developed biofeedback pen, see Figure 1. The shaft of the pen contains a pressure sensor and a red light. When to much hand force was applied on the shaft, the light switched on. All police officers were instructed to follow an instruction protocol. In a standardised way they were asked to loosen the grip when the light switched on during the writing exercise. This ensured a low level of hand and forearm tension.

### Measurement series

In each police officer, we first measured the CROM followed by the v_rad.art.mean_. This sequence was chosen to allow the subjects to settle down and to reduce any possible stress that could influence the measurements. Next, each officer set off for a 2-hour driving task under normal working conditions. Immediately after this task, the CROM and the v_rad.art.mean_ were again measured. After these measurements, the policemen were all asked to write the following line six times: “The weather is fine and the sun is shining” (translated from Dutch) using the biofeedback pen. Finally, after this simple exercise, the CROM and the v_rad.art.mean_ were measured for the last time. To make sure that the conditions were equal for each measurement, the participants were seated on the same chair in upright position. Room temperature and humidity were kept constant as well as other environmental conditions. All measurements were performed by the same investigator to avoid information bias. A hard-copy of all measurements was made for later analysis. During this intervention study, one police officer was unfortunate and fell. He reported acute neck trauma showing restricted neck movement directly after the accident and painful and tense arms in the hours and days following the accident. After a two weeks recovery period, his 1-minute writing exercise was repeated.

### Statistics

Differences in CROM and v_rad.art.mean_ due to the driving and / or the writing task were tested for significance using the paired subject t-test (SPSS, SPSS Institute, Chicago, IL). We pair-wise compared the CROM and v_rad.art.mean_ values after with before driving and after with before writing. Note that the values after the driving task were used as baseline before the writing task. The mean and the 95% confidence intervals (95% CI) of the differences were reported. When this interval does not include zero, the mean of the distribution of means of a parameter is different from zero at p<0.05.

## RESULTS

In all 20 participating male police officers the right arm was the dominant arm. During the 2-hours driving task, each police officer used the left hand for operating the clutch whereas the right hand was used for the throttle. Unfortunately, one police officer had an accident after about half an hour motor driving. This occurred within a few hours after the first CROM and v_rad.art.mean_ measurement. He fell with his motorcycle to the left and reported neck-shoulder complaints with radiation in the left arm, restricted arm movement, light amnesia and vegetative reactions. This incident resulted in a single case with acute neck trauma.

**Figure 2:**
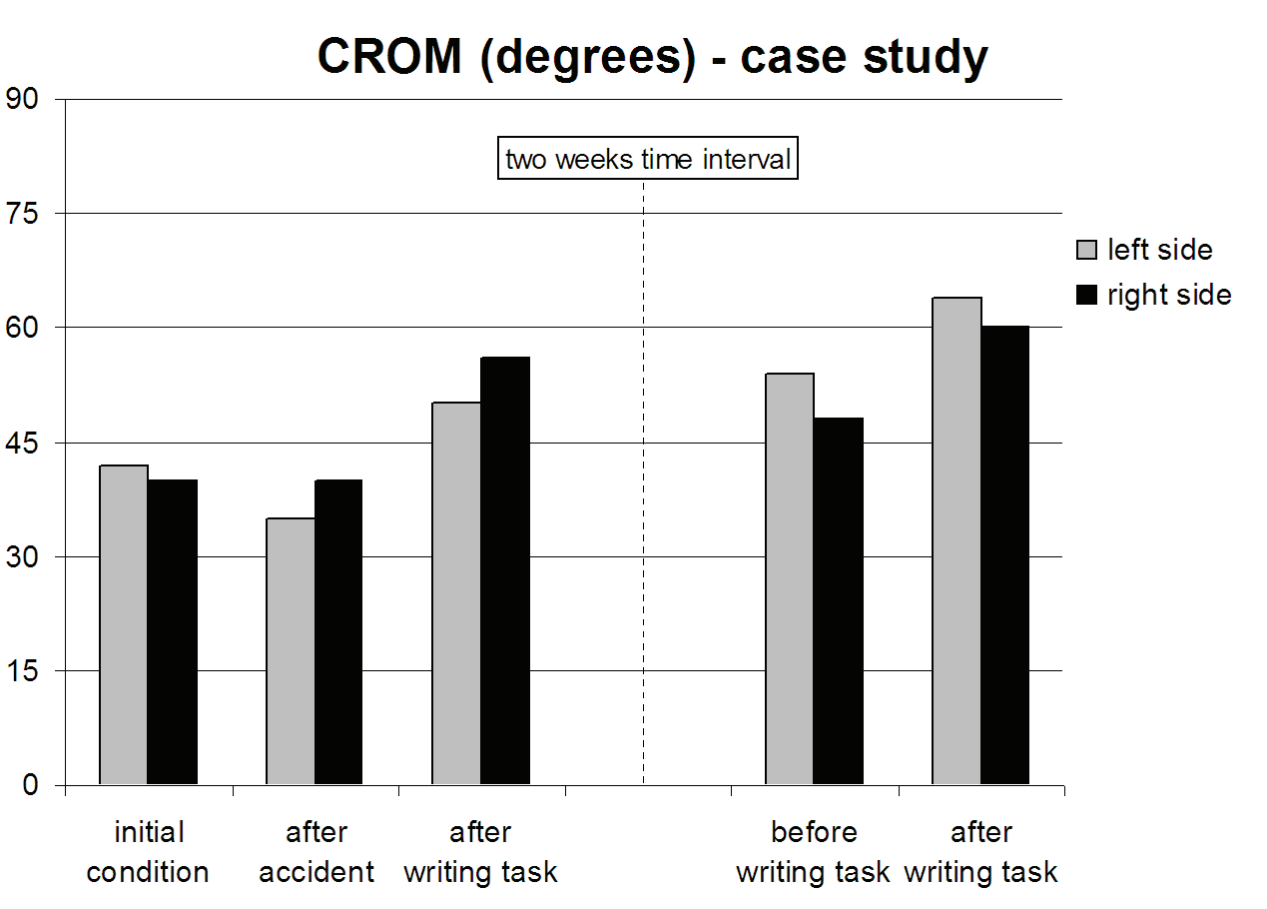
The Cervical Range of Motion (CROM) measured prior to driving (initial condition), immediately after the accident and after the 1-minute writing task using a biofeedback pen. The writing task, aimed at lowering upper extremity tension was repeated after a two week recovery period. After the accident, the CROM decreased to both sides. The CROM increased after the 1-minute writing task and even further increased when this task was repeated after a two weeks recovery period.

**Figure 3:**
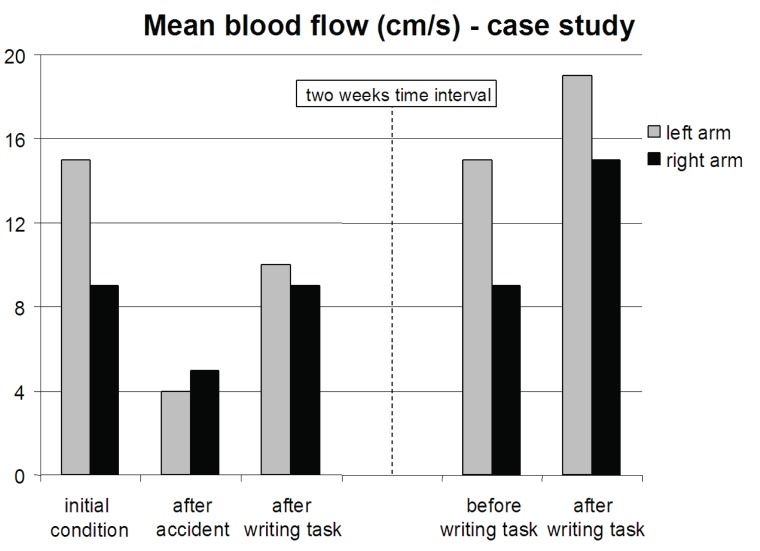
The average radial artery blood flow velocity (v_rad.art.mean_) measured in the left and the right arm. The initial condition showed a higher blood flow in the left arm (used for clutching) compared to the right arm (used for acceleration). After the accident, a significant decrease of blood flow was measured. The blood flow velocity returned to the normal level after the 1-minute writing exercise with a biofeedback pen on the same day. After a two weeks recovery period, baseline blood flow was returned to the normal level and even further increased after 1-minute writing exercise with the biofeedback pen.

Shortly after the accident, the CROM to the left was reduced by 17%, whereas the CROM to the right was unaltered in the single case, see Figure 2. The CROM in the remaining 19 officers showed a significant increase to the right (p<0.05; paired subject t-test), see Table 1. In all subjects, the CROM, on average 52 degrees immediately after the driving task, increased by 20% in both directions after the 1-minute writing exercise with the biofeedback pen (p<0.001). Figure 3 shows the dramatic drop of v_rad.art.mean_ in both arms in the single case after the accident. It reduced by 73% in the right arm and 45% in the left arm. This decrease is in contrast with the values found in the 19 officers after completion of the 2-hours drive, see Table II. No significant changes were found. However, the 1-minute writing exercise increased in the single case the v_rad.art.mean_ by 150% in the right arm and by 80% in the left arm. Also in the group of 19 officers, v_rad.art.mean_ significantly increased by about 50% in each arm after the 1-minute writing exercise using the biofeedback pen (p<0.001).

**Table 1:** Summary of the CROM values

CROM (degrees) (n=19)		

	left side	right side

Initial condition:	51 (47–55)	45 (42–48)
After 2-hours driving task:	53 (50–56)	51 (48–54)
After 1-minute writing task:	62 (59–64)	60 (57–63)
p-value (after versus before driving)	p=0.24	p<0.05
mean (95% CI of the difference)	3 (-2–7)	6 (3–9)
p-value (after versus before writing)	p<0.001	p<0.001
mean (95% CI of the difference)	8 (7–10)	9 (8–11)

Mean (95% Confidence interval) measured in 19 police officers prior to and immediately after the 2-hours motor driving task and after the 1-minute writing task using a biofeedback pen that reduced hand force.

**Table 2:** Summary of the v_rad.art.mean_ values

v_rad.art.mean_ (cm/s) (n=19)		

	left arm	right arm

Initial condition:	8.8 (6.3–11.3)	7.2 (4.2–10.2)
After 2-hours driving task:	9.2 (5.5–13.1)	7.0 (3.8–10.3)
After 1-minute writing task:	12.4 (8.7–16.1)	11.5 (8.0–14.9)
p-value (after versus before driving)	p=0.82	p=0.93
mean (95% CI of the difference)	0.5 (-3.7–4.7)	-0.2 (-4.1–3.8)
p-value (after versus before writing)	p<0.001	p<0.001
mean (95% CI of the difference)	3.2 (1.0–5.3)	4.4 (2.1–6.8)

Mean (95% Confidence interval) measured in 19 police officers prior to and immediately after the 2-hours motor driving task and after the 1-minute writing task using a biofeedback pen that reduced hand force. The mean values and the corresponding 95% Confidence Interval of the differences are listed.

After two weeks of recovery, the CROM and the v_rad.art.mean_ values of the injured police officer returned to levels as assessed before the accident. Both values even improved after the 1-minute writing exercise, see Figures 2 and 3. No significant differences were found between the CROM to the left and the right side and the v_rad.art.mean_ in the left and the right arm prior to and immediately after the driving and writing tasks in the officers group.

## DISCUSSION

This study did not confirm our main hypothesis, that neck mobility and blood flow would decrease after a 2-hours drive on a motorcycle. Based on a self-administered questionnaire, it was previously shown that shoulder stiffness and low-back pain were frequently encountered in motorcycling traffic policemen exposed to occupational vibrations (4). An occupational vibration dose study in this group showed significantly higher prevalence rates for symptoms in fingers and shoulders as compared to the control group (5). We conclude from our study that these symptoms may be the result of chronic exposure to vibrations. We did find modest changes in neck mobility after the 2-hour drive. The cervical range of motion to the right was significantly increased, but no changes in blood flow were measured. The results of our study show, however, that the effect of acute neck trauma can be measured peripherally. This finding was based on a unique measurement prior to and immediately after an unfortunate accident. A fall with abrupt movement of the head whilst wearing a helmet resulted in a decrease of blood flow velocity in both arms. This suggests increased deep neck muscle tension after neck trauma which may be a defence mechanism. With current diagnostic imaging techniques such tension is not visible. In an earlier study, we could image the result of raised neck muscle tension by means of ultrasound applied at the subclavian artery just below the costoclavicular gate (17). The increase of peak blood flow velocity at this site matched with the expected decrease of average blood flow velocity measured peripherally at the radial ventral side of the forearm.

Our secondary hypothesis, that neck mobility and blood flow velocity increase after a biofeedback writing task was confirmed. A subsequent writing task following the driving task showed significant increase in CROM in both directions as well as v_rad.art.mean_ values in both arms. We think that this exercise may initiate an acute decrease in neck, shoulder and especially forearm tension. The latter is supported by a mechanical model on the aetiology of tennis elbow (17). It described the activation of forearm extensors and flexors and their reaction forces in the upper limbs in power gripping and pinching. We hypothesis that relaxation induced in the forearm muscles induces scalene muscle relaxation as well, thereby increasing blood flow velocity and neck mobility by decompression of the scalene triangle. Another explanation could be that forearm relaxation affected vasoconstriction, i.e. that it induced vasodilatation in the forearm arteries and veins. This mechanism could also have increased blood flow velocity, but it cannot clarify the increased neck mobility. In the case of the motor driver who fell, we clearly measured a decreased neck mobility and blood flow. This was most probably the result of post-traumatic defensive neck tension since the remaining 19 officers did not show decrease in blood flow velocity after the intervention. The biofeedback writing task resulted in acute improvement of neck mobility and blood flow velocity in both arms in this police officer. Clearly, the two-week rest period after the accident had positive effects on the CROM and the blood flow velocity. These parameters returned to the same values as before the driving task. But still, after two weeks of rest, comparison before and immediately after the biofeedback writing task again showed improvement of CROM and v_rad.art.mean_ values.

Although actual measurement values quantified neck mobility and blood flow velocity in our test group, the study setup was an observational study. We therefore did not include a control group, which is one of the study limitations. Especially the result on biofeedback writing must be interpreted with great care. Without a control group writing with a standard pen or writing not at all for one minute, the increase in neck mobility and blood flow velocity cannot simply be ascribed to ‘biofeedback’. It may well be the explanatory factor, but a randomised control trial has to be done to test this aspect on muscle relaxation and diverting attention. Another limitation was the controlling of the intervention. Although a driving task was done under normal working conditions, it is impossible to state that the intervention was similar for each police officer.

It is known that thoracic outlet syndrome (TOS) develops between 30-50 years old of life (2, 18). Two main groups can be identified, i.e. neurologic TOS (describing up to 97% of cases and vascular TOS (arterial or venous) (19). Although vascular TOS is identified in a minority of cases, our results show that writing with biofeedback might improve decompression of the subclavian artery passing through the interscalene triangle. More research is necessary on this decompressing effect in vascular as well as neurologic TOS. We therefore suggest that a simple writing exercise based on biofeedback of hand force might has potential therapeutic effects on the management of general TOS and work-related upper extremity disorders (20, 21, 22).
